# Development and validation of a prognostic scoring model for mortality risk stratification in patients with recurrent or metastatic gastric carcinoma

**DOI:** 10.1186/s12885-021-09079-7

**Published:** 2021-12-12

**Authors:** Tai Ma, Zhijun Wu, Xiaopeng Zhang, Hui Xu, Ying Feng, Cheng Zhang, Minmin Xie, Yahui Yang, Yi Zhang, Chong Feng, Guoping Sun

**Affiliations:** 1 Department of Oncology, The First Affiliated Hospital of Anhui Medical University, 218 Jixi Road, Hefei, Anhui 230022 People’s Republic of China; 2 Department of Oncology, Ma’anshan Municipal People’s Hospital, Ma’anshan, Anhui 243000 People’s Republic of China; 3 Department of Non-communicable Diseases and Health Education, Hefei Center for Disease Control and Prevention, Hefei, Anhui 230061 People’s Republic of China; 4 Anhui Provincial Cancer Institute/Anhui Provincial Office for Cancer Prevention and Control, Hefei, Anhui 230022 People’s Republic of China

**Keywords:** Stomach neoplasms, Neoplasm metastasis, Survival analysis, Nomograms

## Abstract

**Background:**

Survival times differ among patients with advanced gastric carcinoma. A precise and universal prognostic evaluation strategy has not yet been established. The current study aimed to construct a prognostic scoring model for mortality risk stratification in patients with advanced gastric carcinoma.

**Methods:**

Patients with advanced gastric carcinoma from two hospitals (development and validation cohort) were included. Cox proportional hazards regression analysis was conducted to identify independent risk factors for survival. A prognostic nomogram model was developed using R statistics and validated both in bootstrap and external cohort. The concordance index and calibration curves were plotted to determine the discrimination and calibration of the model, respectively. The nomogram score and a simplified scoring system were developed to stratify patients in the two cohorts.

**Results:**

Development and validation cohort was comprised of 401 and 214 gastric cancer patients, respectively. Mucinous or non-mucinous histology, ECOG score, bone metastasis, ascites, hemoglobin concentration, serum albumin level, lactate dehydrogenase level, carcinoembryonic antigen level, and chemotherapy were finally incorporated into prognostic nomogram. The concordance indices were 0.689 (95% CI: 0.664 ~ 0.714) and 0.673 (95% CI: 0.632 ~ 0.714) for bootstrap and external validation. 100 and 200 were set as the cut-off values of nomogram score, patients in development cohort were stratified into low-, intermediate- and high-risk groups with median overall survival time 15.8 (95% CI: 12.2 ~ 19.5), 8.4 (95% CI: 6.7 ~ 10.2), and 3.9 (95% CI: 2.7 ~ 5.2) months, respectively; the cut-off values also worked well in validation cohort with different survival time in subgroups. A simplified model was also established and showed good consistency with the nomogram scoring model in both of development and validation cohorts.

**Conclusion:**

The prognostic scoring model and its simplified surrogate can be used as tools for mortality risk stratification in patients with advanced gastric carcinoma.

**Supplementary Information:**

The online version contains supplementary material available at 10.1186/s12885-021-09079-7.

## Background

The survival of patients with recurrent or metastatic gastric cancer is poor. According to an analysis of population-based data in the United States, more than a third of gastric cancer patients were metastatic at diagnosis [1]. The five-year relative survival rate for these patients was only 5.3% [1]. Furthermore, data from a cancer registry in Shanghai, China showed that the five-year survival rate of stage IV gastric cancer diagnosed between 2002 and 2003 was not more than 10%, with a median survival time of approximately 8 months [2]. Although therapeutic efforts have been exerted in recent years, the median survival time has remained approximately 8–14 months [3–10].

Prognosis is different among gastric cancer patients with distant metastatic disease. Several clinical, pathological, molecular, and genetic variables were identified as prognostic factors in different studies. In view of its clinical applicability, a simple and reliable prognosis stratification tool provides significant value in the clinical management of patients. Instead of molecular or genetic variations, variables derived from routine clinical data can be integrated into prognostic models. As early as 2004, Chau et al. [11] developed a four-factor prognostic model that incorporated performance status, liver metastases, peritoneal metastases, and alkaline phosphatase levels. In this prognostic model, advanced gastric cancer patients were distinctly stratified into three risk groups. Subsequently, several other prognostic models were constructed, but they were limited in terms of applicability or credibility. We performed a prognostic model research using two isolated datasets of patients derived from two different cohorts of Chinese patients to create a scoring system and tried to stratify advanced gastric cancer patients into different prognostic subgroups.

## Methods

### Patient selection and data collection

**Fig. 1:**
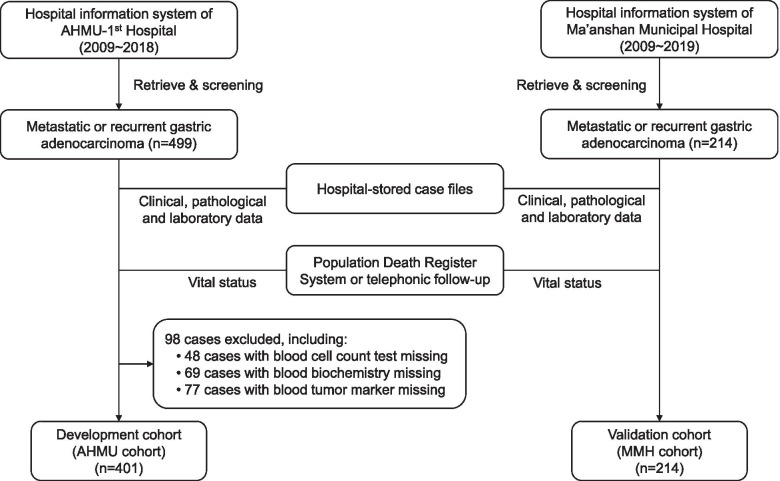
Diagram of patient selection and data collection. AHMU: Anhui Medical University (Anhui Province, China); MMH: Ma’anshan Municipal People’s Hospital (Anhui Province, China)

The data used for model development and validation were derived from two cancer patient cohorts from two different hospitals in Anhui Province, People’s Republic of China, the First Affiliated Hospital of Anhui Medical University (AHMU) and the Ma’anshan Municipal People’s Hospital (MMH), respectively. The approach and procedure of the study were approved by the ethics committee of the First Affiliated Hospital of AMHU and the MMH. A diagram of patient selection and data collection is presented in Fig. 1.

The gastric cancer patient list was retrieved from the hospital information system. The criteria for candidate selection included: (1) histopathologically confirmed gastric or esophagogastric junction carcinoma; (2) distant metastatic disease irrespective of the primary staging; and (3) distant metastasis diagnosed between 2009 and 2018 in the AHMU cohort and between 2009 and 2019 in the MMH cohort. Patients with multiple primary cancers were excluded from the study. The stored case files were then reviewed.

Essential clinical, pathological, and laboratory information such as the following was extracted from the document: (1) patient-related characteristics such as age, sex, performance status during the first appearance of metastasis, previous gastrectomy, systemic treatment and local treatment; (2) tumor-related variables, including WHO histology, primary staging at the time of diagnosis, tumor grade, Her-2 status, date when the first episode of metastasis appeared, metastatic site(s), and number of metastatic organs at the first episode of metastasis; (3) results of routine laboratory tests such as blood count, serum biochemistry, and tumor markers during the first episode of metastatic disease occurrence, wherein tests were conducted before any metastasis-aimed anti-cancer therapy and within a 7-day interval from radiologically documented distant metastasis.

### Endpoint and follow-up

Death due to any cause was considered as the endpoint in the current study. All patients enrolled in the study were matched in the death registry system, which was developed by the Chinese Center for Disease Control and Prevention. The date and cause of death were documented. For unmatched patients, vital status was followed-up through telephone communication with the patients or with their relatives. Survival time in months was calculated using the death date or last follow-up date and the date when the first episode of metastasis occurred.

### Screening for prognostic factors

To be practicable in models, candidate prognostic factors should be easily measurable, stable, and widely applicable. In addition to clinical and pathological parameters, several blood indices were obtained. These indices included hemoglobin (HGB) concentration, platelet count in blood count test, albumin (ALB), and lactate dehydrogenase (LDH) levels in serum biochemistry, and serum carcinoembryonic antigen (CEA) levels in tumor marker tests. Numerical variables were transformed into categorical variables. The X-tile software version 3.6.1 (Rimm Lab, Yale University) was used to plot the best cut-off values in terms of their impact on survival [12]. The Univariate Cox regression was used in the primary screening of prognostic factors in the development cohort (AHMU cohort). Statistically and clinically significant variables were included in the multivariate Cox hazard model analysis. Cox regression was performed using the SPSS 22.0 statistical software (IBM Corp., Armonk, NY, USA). The forward stepwise method was used to select prognostic predictive variables, including parameters with *P* values < 0.05, and excluding those with *P* values > 0.10. The Cox regression results were described as hazard ratios (HRs) and 95% confidence intervals (CIs). All *P* values were 2-tailed. *P* values less than 0.05 were considered statistically significant.

### Construction and validation of the Nomogram scoring model

The nomogram model was built and validated according to methods described before [13]. The nomogram was plotted using the “nomogram” function in the ‘R’ version 4.0.3 (The R Foundation for Statistical Computing, Vienna, Austria) with the ‘rms’ and ‘survival’ packages (http://www.r-project.org/). Discrimination and calibration were used to assess the accuracy of the nomogram model. Discrimination is the ability of the model to separate patients according to their survival status. It was reflected by the calculated Harrell concordance index (c-index). Calibration refers to the discrepancy between predictions and actual survival outcomes. It was measured by graphic calibration curves that represented the relationship between the observed outcome frequencies and predicted probabilities. Calibration curves were plotted to compare the nomogram-predicted 3-month, 6-month, and 12-month survival probabilities with the observed survival outcomes. The validation procedures were also performed using the ‘R’ version 4.0.3. For the internal validation of the nomogram model, 1000 bootstraps with sample sizes of 120 were generated from the AHMU cohort. The external validation dataset included patients in the MMH cohort, and 1000 bootstrapping (size 60) was performed to calculate and plot the calibration curves.

### Simplification and application of prognostic scoring model

To verify the prognosis-distinguishment ability of the nomogram scoring model in gastric cancer patients, the total score of each patient in the development cohort was calculated. The best cut-off values of the total score were determined using the X-tile software with adjustment. Patients in the development and validation cohorts were stratified into high-, intermediate-, and low-risk death groups. Simplified scores were then allocated to each patient according to the presence (1 point) and absence (0 point) of high-risk variables in the nomogram. As to three-categorical ordinal variables, 0, 1, and 2 points were allocated for each risk strata, respectively. The sum of the total simplified score of each patient was calculated. In accordance with the total score, the cut-off values of the total simplified score were determined by the X-tile software with adjustment. Patients in the development cohort were divided into high-, intermediate-, and low-risk groups, and the same classification algorithm was used in the validation cohort. The Log-rank test with pairwise comparisons in the Kaplan-Meier survival analysis was used to compare the survival times of different risk groups. *P* value of < 0.05 was considered statistically significant. Survival analysis was performed using the SPSS 22.0 statistical software (IBM Corp., Armonk, NY), and survival curves were drawn using the GraphPad Prism software version 5.01 (GraphPad Software, Inc., San Diego, CA, USA).

## Results

### Patient characteristics

At first, 499 and 214 eligible cases were identified in the hospital information system of AHMU and MMH respectively. After reviewing the case files, 98 cases in the AHMU cohort were excluded from analysis due to missing essential blood tests results. Finally, a total of 615 gastric cancer patients (401 in the AHMU cohort and 214 in the MMH cohort) was recruited to participate in the study. All patients had pathologically diagnosed carcinoma with radiological or pathological evidence of distant metastatic disease. The AHMU cohort included 169 patients with primary metastasis (metastatic gastric cancer) and 232 patients with metastasis after a postoperative disease-free interval (recurrent gastric cancer). All metastatic diseases were diagnosed between June 2, 2009, and May 10, 2018. By the last follow-up that occurred on August 1, 2018, 351 cases died, and 50 cases were still alive. In the MMH cohort, there were 100 cases of primary metastasis and 114 cases of postoperative metastasis. Metastatic diseases were diagnosed between August 15, 2009, and December 13, 2019. By November 10, 2020, 206 patients died, while 8 cases were still alive. The median overall survival (mOS) for patients in the AHMU and MMH cohorts were 11.0 (95% CI: 9.6 ~ 12.4) and 6.5 (95% CI: 5.3 ~ 7.7) months, respectively.

Table 1 and Additional file 1 shows the distribution of the patients’ clinical parameters and laboratory tests. In the MMH cohort, in which 35.0% were 70 years old or above, there were more elderly patients. The median age in the MMH cohort was 66 years (vs. 61 years in the AHMU cohort, Wilcoxon W test, *P* < 0.001). The performance status (PS) was also poorer in the MMH cohort wherein 41.6% of the patients had a PS of two or above. However, in the AHMU cohort, only 20.4% of the patients were assessed as having a PS of two or above at the first appearance of metastatic foci (Pearson χ^2^ test, *P* < 0.001). Accordingly, hemoglobin, serum albumin levels, and platelet counts were also lower in the MMH cohort. There were no differences in serum LDH and CEA levels between the two cohorts. For the metastatic sites, liver metastasis accounted for 31.2 and 42.1% of the patients in the AHMU and MMH cohorts, respectively (Pearson χ^2^ test, *P* = 0.007). The frequency of lung metastasis and distant lymph node metastasis were also higher in the MMH cohort than those in the AHMU cohort (lung metastasis: 33.7% vs. 10.0%; distant lymph node metastasis: 63.1% vs. 54.6%, Pearson χ^2^ test, *P* < 0.001). The distribution of bone metastasis and peritoneal metastasis in the two cohorts were uniform. At the first appearance of metastatic disease, 76.8% of patients in the AHMU cohort were diagnosed with single organ involvement, while 53.7% were diagnosed in the MMH cohort. Nearly half (46.3%) of the patients presented with two or more sites of metastasis. In addition, 72.0% of the tumors in the MMH cohort were poorly differentiated (G3) or undifferentiated (G4). This was higher than the proportion found in the AHMU cohort (58.1%, Pearson χ^2^ test, *P* = 0.001). After metastasis was confirmed, 70.1 and 86.5% of the patients in the MMH and AHMU cohorts, respectively, received palliative chemotherapy. The difference was statistically significant (Pearson χ^2^ test, *P* < 0.001).

**Table 1:** Baseline characteristics of gastric cancer patients in AHMU (development) cohort and MMH (validation) cohort

Clinical characteristics	AHMU cohort [*n (%)*, *n* = 401]	MMH cohort [*n (%)*, *n* = 214]	*P*
Age (years) (*median, P25 ~ 75*)	61, 53 ~ 68	66, 57 ~ 73	< 0.001
< 70	318 (79.3)	139 (65.0)	< 0.001
70 ~	83 (20.7)	75 (35.0)	
Sex			0.083
Male	267 (66.6)	157 (73.4)	
Female	134 (33.4)	57 (26.6)	
Synchronous or metachronous metastasis			0.275
Synchronous	169 (42.1)	100 (46.7)	
Metachronous	232 (57.9)	114 (53.3)	
Gastrectomy			0.976
None	150 (37.4)	82 (38.3)	
Curative	211 (52.6)	111 (51.9)	
Palliative	40 (10.0)	21 (9.8)	
ECOG score at first episode of metastasis			< 0.001
0 ~ 1	319 (79.6)	125 (58.4)	
2 ~	82 (20.4)	89 (41.6)	
WHO histology			0.352
Non-mucinous adenocarcinoma	362 (90.3)	198 (92.5)	
Mucinous adenocarcinoma	39 (9.7)	16 (7.5)	
Tumor grade			0.001
G1–2	70 (17.5)	33 (15.4)	
G3–4	233 (58.1)	154 (72.0)	
Unknown	98 (24.4)	27 (12.6)	
Metastatic to (first episode of metastasis)			
Liver	125 (31.2)	90 (42.1)	0.007
Lung	40 (10.0)	70 (32.7)	< 0.001
Bone	28 (7.0)	21 (9.8)	0.217
Distant lymph node	219 (54.6)	135 (63.1)	0.043
Peritoneal/Malignant ascites	62 (15.5)	40 (18.7)	0.305
Number of involved organs in first episode of metastasis			< 0.001
1	308 (76.8)	115 (53.7)	
2 ~	93 (23.2)	99 (46.3)	
Resection of metastatic tumor			0.088
Yes	8 (2.0)	0 (0.0)	
No	393 (98.0)	214 (100.0)	
Palliative chemotherapy			< 0.001
With	347 (86.5)	150 (70.1)	
Without	54 (13.5)	64 (29.9)	
Blood test during first episode of metastasis			
HGB level (g/L) (*median, P25 ~ 75*)	113.0, 98.0 ~ 123.0	108.5, 88.0 ~ 122.0	0.022
< 90	62 (15.5)	55 (25.7)	0.002
90 ~	339 (84.5)	159 (74.3)	
Platelet count (×10^9^/L) (*median, P25 ~ 75*)	200, 148 ~ 278	193, 136 ~ 252	0.035
< 300	325 (81.0)	189 (88.3)	0.020
300 ~	76 (19.0)	25 (11.7)	
ALB level (g/L) (*mean, SD*)	38.2, 5.1	36.2, 5.9	< 0.001
< 38	179 (44.6)	130 (60.7)	< 0.001
38 ~	222 (55.4)	84 (39.3)	
LDH level (U/L) (*median, P25 ~ 75*)	183, 153 ~ 243	178, 147 ~ 242	0.443
< 220	281 (70.1)	145 (67.8)	0.553
220 ~	120 (29.9)	69 (32.2)	
CEA level (ng/mL) (*median, P25 ~ 75*)	5.1, 2.0 ~ 36.4	6.2, 2.7 ~ 33.1	0.142
< 8	225 (56.1)	119 (55.6)	0.862
8 ~	115 (28.7)	59 (27.6)	
100 ~	61 (15.2)	36 (16.8)	

^a^*Z* value by nonparametric Wilcoxon W test; ^b^Continuity Correction χ2; ^c^*t* value by independent sample T test

*AHMU* Anhui Medical University (Anhui Province, China), *MMH* Ma’anshan Municipal People’s Hospital (Anhui Province, China); *P25 ~ 75*: upper quartile to lower quartile, *ECOG* The Eastern Cooperative Oncology Group, *HGB* Hemoglobin, *ALB* Albumin, *LDH* Lactate dehydrogenase, *CEA* Carcinoembryonic antigen

### Survival-related prognostic factors in metastatic gastric Cancer patients

Several continuous parameters were transformed into categorical variables before their introduction into the Cox regression equation in consideration of statistical analysis and clinical practice. The best cut-off values were automatically determined using the X-tile software. With regards to the age at first metastasis, the largest survival difference was observed while dividing age at 70 years. As shown in Table 2, patients older than 70 years showed an increase in mortality hazard compared to that in patients younger than 70 years (*HR* = 1.34, 95% CI: 1.04 ~ 1.72, *P* = 0.024). Accordingly, HGB concentration was divided into the “< 90 g/L” and “≥ 90 g/L” groups. Platelet count was categorized into the “< 300 × 10^9^/L” and ≥ 300 × 10^9^/L” groups. ALB and LDH levels were also divided into two binary categorical variables with cut-off values of 38 and 220, respectively. Patients with HGB < 90 g/L, platelet count ≥300 × 10^9^/L, ALB < 38 g/L, or LDH ≥ 300 U/L had a higher risk of death than that in their counterparts. CEA level was introduced into the Cox analysis as a triple-categorical covariate: “< 8 ng/mL”, “8 ng/mL ~” and “≥ 100 ng/mL”. Patients with higher CEA levels had poorer survival. The univariate Cox regression also revealed that patients with Eastern Cooperative Oncology Group (ECOG) score of ≥2, poorly differentiated or undifferentiated tumors, bone metastasis, and peritoneal metastasis or malignant ascites at the first episode of metastasis had a higher risk of death. Palliative chemotherapy significantly decreased the risk for death (hazard ratio [HR], 0.71; 95% confidence interval [CI], 0.52 ~ 0.97; *P* = 0.030).

**Table 2:** Cox proportion hazard model analysis for death risk in gastric cancer patients in development cohort

Variables	Univariate Cox regression				Multivariate Cox regression (Forward Stepwise: LR)			
	*β*	*HR*	*95% CI*	*P*	*β*	*HR*	*95% CI*	*P*
Age (years), (“< 70” as reference)								
70 ~	0.291	1.34	1.04 ~ 1.72	0.024	–	–	–	0.917
Sex, (“Male” as reference)					Not included			
Female	−0.030	0.97	0.78 ~ 1.21	0.790				
Synchronous or metachronous metastasis, (“Metachronous” as reference)								
Synchronous	0.175	1.19	0.96 ~ 1.47	0.105	–	–	–	0.666
Gastrectomy, (“None” as reference)								
Curative	−0.376	0.69	0.55 ~ 0.86	0.001	–	–	–	0.065
Palliative	−0.103	0.90	0.63 ~ 1.30	0.578	–	–	–	0.634
ECOG score, (“0 ~ 1” as reference)								
2 ~	0.495	1.64	1.27 ~ 2.11	< 0.001	0.322	1.38	1.05 ~ 1.81	0.021
WHO histology (“Non-mucinous” as reference)								
Mucinous adenocarcinoma	0.429	1.54	1.09 ~ 2.16	0.014	0.590	1.80	1.27 ~ 2.56	0.001
Tumor grade, “G1–2” as reference								
G3–4	0.489	1.63	1.21 ~ 2.20	0.001	–	–	–	0.058
Unknown	0.390	1.48	1.05 ~ 2.07	0.024	–	–	–	0.499
Metastatic to, (“Absent” as reference)								
Liver	0.134	1.14	0.91 ~ 1.43	0.239	–	–	–	0.433
Lung	−0.410	0.66	0.46 ~ 0.95	0.026	–	–	–	0.156
Bone	0.742	2.10	1.41 ~ 3.12	< 0.001	0.561	1.75	1.16 ~ 2.66	0.008
Distant lymph node	−0.091	0.91	0.74 ~ 1.13	0.395	–	–	–	0.922
Peritoneal/Malignant ascites	0.405	1.50	1.13 ~ 2.00	0.006	0.532	1.70	1.26 ~ 2.30	0.001
No. of metastatic organ(s), (“1” as reference)					Not included			
2 ~	0.188	1.21	0.94 ~ 1.54	0.136				
Resection of metastatic tumor, (“No” as reference)								
Yes	0.054	1.06	0.50 ~ 2.23	0.887	–	–	–	0.552
Palliative chemotherapy, (“Without” as reference)								
With	−0.339	0.71	0.52 ~ 0.97	0.033	−0.377	0.69	0.50 ~ 0.94	0.020
HGB concentration (g/L), (“< 90” as reference)								
90 ~	−0.342	0.71	0.54 ~ 0.94	0.016	−0.337	0.71	0.53 ~ 0.96	0.024
Platelet count (×10^9^/L) (“< 300” as reference)								
300 ~	0.347	1.42	1.09 ~ 1.84	0.009	–	–	–	0.116
ALB level (g/L), (“< 38” as reference)								
38 ~	−0.527	0.59	0.48 ~ 0.73	< 0.001	−0.400	0.67	0.54 ~ 0.84	< 0.001
LDH level (U/L), (“< 220” as reference)								
220 ~	0.598	1.82	1.45 ~ 2.28	< 0.001	0.532	1.70	1.33 ~ 2.18	< 0.001
CEA level (ng/mL), (“< 8” as reference)								
8 ~	0.365	1.44	1.13 ~ 1.83	0.003	0.288	1.33	1.04 ~ 1.72	0.024
100 ~	0.828	2.29	1.69 ~ 3.09	< 0.001	0.752	2.12	1.54 ~ 2.93	< 0.001

*LR* Likelihood Ratio, *HR* Hazard ratio, *95% CI* 95% confident interval, *ECOG* The Eastern Cooperative Oncology Group, *HGB* Hemoglobin, *ALB* Albumin, *LDH* Lactate dehydrogenase, *CEA* Carcinoembryonic antigen

We then selected covariates with statistical significance in the univariate Cox analysis and those who did not show statistical significance but were considered as clinically meaningful. These selected covariates were included in the multivariate Cox model. After a forward stepwise analysis, nine variables were retained in the equation. These variables were as follows: Mucinous or non-mucinous histology, ECOG score, bone metastasis, peritoneal metastasis or malignant ascites, HGB concentration, ALB level, LDH level, CEA level, and palliative chemotherapy. These were considered as independent prognostic factors of metastatic gastric cancer. The β coefficients of the Cox equation, HRs, and *P* values are shown in Table 2.

### Nomogram model and validation

**Fig. 2:**
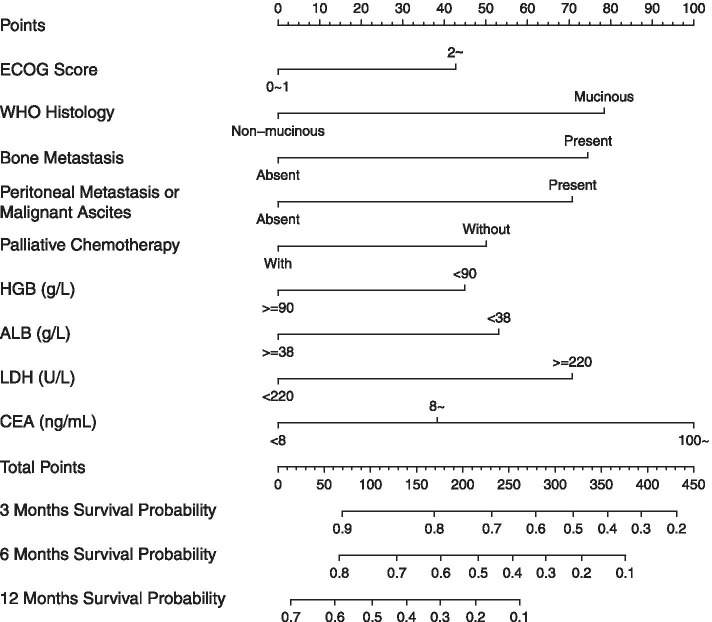
Nomogram of survival prediction in metastatic or recurrent gastric carcinoma patient. HGB: hemoglobin; LDH: lactate dehydrogenase; ALB: albumin; CEA: carcinoembryonic antigen; ECOG: The Eastern Cooperative Oncology Group

After running the R program, a prognostic nomogram was plotted based on the β coefficients of the forementioned nine parameters (Fig. 2). In this nomogram, for individuals with metastatic or recurrent gastric cancer, 3-month, 6-month, and 12-month survival probabilities can be predicted according to their characteristics at the first episode of metastasis. Table 3 shows the detailed rules of scoring point assignment. For instance, an initial stage IV gastric non-mucinous (0 point) cancer patient presented with liver metastasis (0 point), his or her ECOG score was 2 (43 points), HGB was 80 g/L (45 points), LDH was 534 U/L (71 points), ALB was 35 g/L (53 points), CEA was 6 ng/mL (0 point), and no palliative chemotherapy was administered (50 points). The total score was 262 for this patient. The 3-, 6-, and 12-month survival probabilities were slightly greater than 0.6, around 0.5, and around 0.1, respectively.

**Table 3:** Rules of scoring points assignment in β Coefficient-based and simplified prognostic scoring models

Parameters	β coefficient-based (nomogram) score	Simplified score
ECOG score		
0 ~ 1	0	0
2 ~	43	1
WHO histology		
Non-mucinous adenocarcinoma	0	0
Mucinous adenocarcinoma	78	1
Bone metastasis		
Present	75	1
Absent	0	0
Peritoneal metastasis/Malignant ascites		
Present	71	1
Absent	0	0
Palliative chemotherapy		
Without	50	1
With	0	0
HGB level (g/L)		
< 90	45	1
≥ 90	0	0
ALB level (g/L)		
< 38	53	1
≥ 38	0	0
LDH level (U/L)		
< 220	0	0
≥ 220	71	1
CEA level (ng/mL)		
< 8	0	0
8 ~	38	1
100 ~	100	2

*ECOG* The Eastern Cooperative Oncology Group, *HGB* Hemoglobin, *ALB* Album, *LDH* Lactate dehydrogenase, *CEA* Carcinoembryonic antigen

**Fig. 3:**
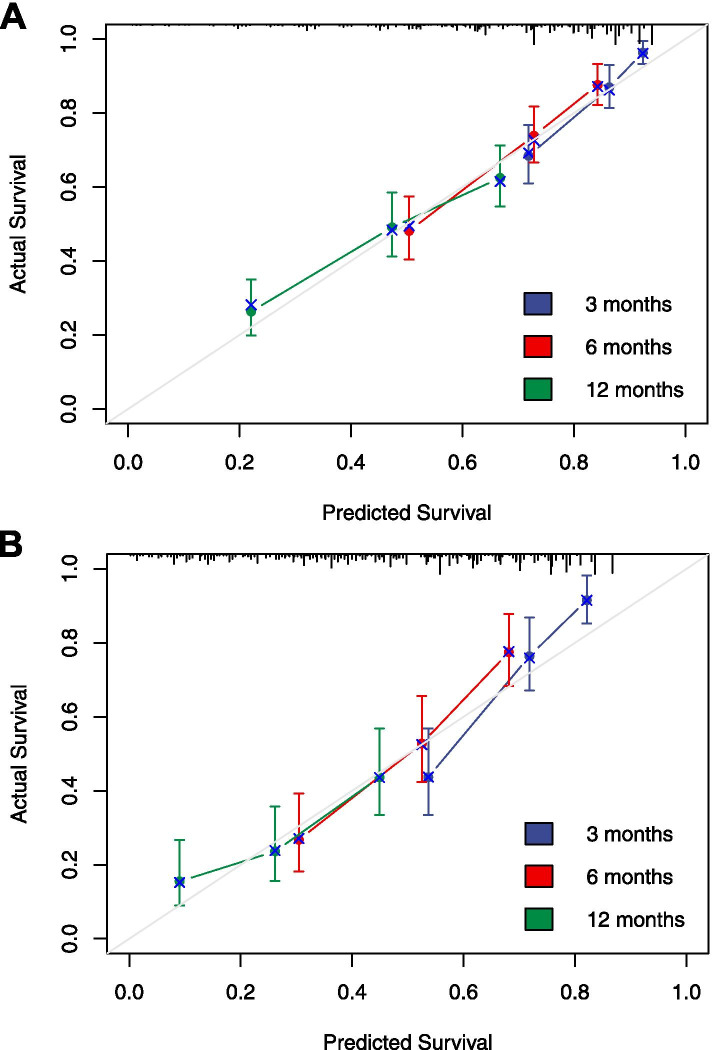
Calibration curves of the prognostic predicting model for patients with metastatic or recurrent gastric cancer carcinoma. **A** Inner bootstrap validation in AHMU cohort for 3-, 6- and 12-month survival. 1000 times bootstrap with sample size 120 subjects per group. **B** External validation of 3-, 6- and 12-month survival using the MMH cohort of 214 patients, with samples sizes of 60. The 45^o^ grey lines show the ideal reference lines where the predicted survival probabilities match the actual survival proportions. Dots indicate the predicted probabilities for the resampled groups of patients with their respective 95% confidence intervals

To validate the nomogram, bootstrap and external validation in the MMH cohort were used. The c-indices of the nomogram by bootstrap and external validation were 0.689 (95% CI: 0.664 ~ 0.714) and 0.673 (95% CI: 0.632 ~ 0.714), respectively. Figure 3 shows the calibration curves for bootstrap validation and external validation. The x-axes represent the predicted 3-, 6-, and 12-month survival probabilities by nomogram in the AHMU cohort and MMH cohort. The y-axes represent actual survival and discrepancy between predictions and actual survival outcomes, which can be reflected by the deviation from the grey 45^o^ lines (ideal situation). The results indicated moderate discrimination and good calibration of the model in both the inner and external validation cohorts.

### Patient stratification and simplified scoring model

**Fig. 4:**
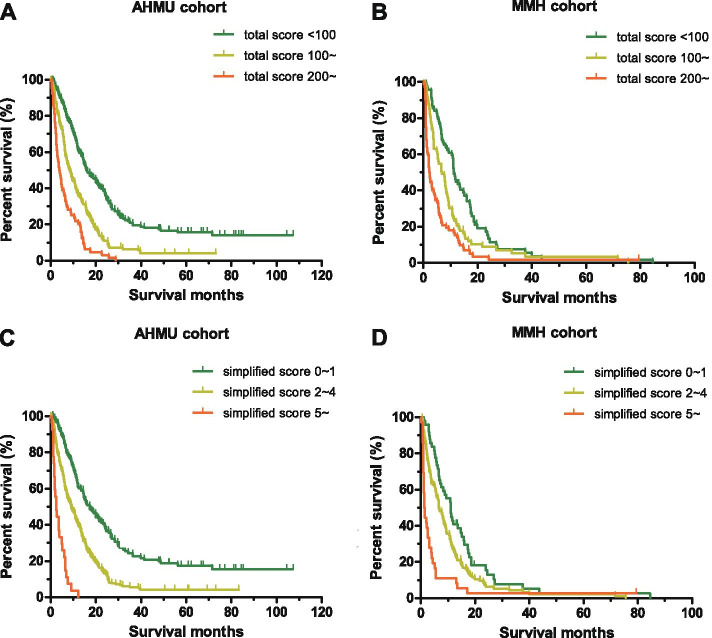
The Kaplan-Meier survival curves for metastatic or recurrent gastric carcinoma patients with different scores. The log-rank test method with pairwise comparisons was used to compare survival times among the different risk subgroups. Total scores were calculated according to the prognostic nomogram. Cut-off values of 100 and 200 were used to divide patients in the AHMU cohort (**A**) and the MMH cohort (**B**); Patients in the AHMU cohort (**C**) and the MMH cohort (**D**) were divided into three groups according to the simplified score of 0 ~ 1, 2 ~ 4, and 5~. AHMU: Anhui Medical University (Anhui Province, China); MMH: Ma’anshan Municipal People’s Hospital (Anhui Province, China)

All cases in the development cohort were scored using the β coefficient-based (nomogram) scoring rules according to the characteristics of the nine parameters. Additional file 2 showed the distribution of total score in patients of the two cohorts. The X-tile soft initially generated 91 and 171 as cut-off values of the total score, with a maximized χ^2^ in the log-rank test. However, it failed to separate low- and intermediate-risk groups in the validation cohort. We adjusted them to 100 and 200, then yielded an acceptable χ^2^ value. This also worked well in the validation cohort. A total of 401 patients in the AHMU cohort was divided into three subgroups: the “total score < 100”, the “total score ≥ 100 but < 200” and the “total score ≥ 200”, with a sample size of 184, 153, and 64 in each subgroup, respectively. As showed in Fig. 4A, the Kaplan-Meier estimated survival curves were separated clearly with statistical significance. The corresponding median OS for the three subgroups were 15.8 (95% CI: 12.2 ~ 19.5), 8.4 (95% CI: 6.7 ~ 10.2), and 3.9 (95% CI: 2.7 ~ 5.2) months, respectively. All *P* values were < 0.001 in pairwise comparisons of the log-rank test. While applying the same algorithms and grouping rules to patients in the MMH validation cohort, we also obtained separated survival curves (Fig. 4B). The median OS were 11.2 (95% CI: 10.2 ~ 12.1) months in patients with a total score of < 100 (low-risk), 6.7 (95% CI: 4.3 ~ 9.0) months in the “total score 100 ~ 200” group (intermediate-risk), and 2.2 (95% CI: 1.6 ~ 2.8) months in the “total score 200 ~” group (high-risk) (log-rank test, *P* < 0.05). To date, a mortality risk stratification method for advanced gastric cancer has been successfully developed. The exemplified patient above whose total score was 262 could be stratified into the high-risk subgroup with the poorest prognosis.

We further simplified the 9- parameter nomogram scoring model for convenience in clinical practice. The rule of scoring point assignment is shown in Table 3. The total simplified scores for patients in the development cohort were calculated and were showed in Additional file 2. Finally, patients in the AHMU cohort were separated into low- (score 0 ~ 1), intermediate- (score 2 ~ 4), or high-risk (score 5 and more) subgroups, with median OS of 17.4 (95% CI: 13.2 ~ 21.4), 9.2 (95% CI: 7.0 ~ 11.4), and 2.4 (95% CI: 1.3 ~ 3.5) months, respectively (log-rank test, *P* < 0.001). These digital numbers were comparable to those in the above subgroups, which were divided by the total nomogram scores. Figure 4C shows the survival curves for the three groups. The trend of lines was parallel to those in Fig. 4A. In addition, this simplified scoring rule also worked well in the 214-patient validation cohort with perfectly separated survival curves (Fig. 4D). The mOS for the low-, intermediate-, and high-risk groups in the MMH cohort were 11.0 (95% CI: 8.4 ~ 13.6), 6.7 (95% CI: 5.1 ~ 8.2), and 1.3 (95% CI: 0.9 ~ 1.7) months, respectively (log-rank test, *P* < 0.05). The simplified model was considered as a surrogate for the nomogram scoring model in stratifying patients with advanced gastric cancer based on their mortality risk evaluation. The above-exemplified patient with a simplified score of 5 was classified into the high-risk subgroup, which was almost identical to aforementioned grouping.

## Discussion

The survival of patients with metastatic or recurrent gastric cancer is influenced by several factors. This study generated a prognostic nomogram (the AHMU scoring model) involving nine independent prognostic factors for these patients. One patient-related parameter, the ECOG performance status at the first onset of metastasis; three tumor-related parameters including WHO histology, bone metastasis and peritoneal metastasis or malignant ascites in the first episode of metastasis; four laboratory-related parameters including HGB concentration, ALB level, LDH level, CEA level; and one treatment-related parameter, palliative chemotherapy were included. All variables were obtained easily and quickly through routine clinical inspections. In fact, there have been several other prognostic scoring or stratifying models for advanced gastric or esophagogastric cancer published in the last 10 years, of which the Royal Marsden Hospital (RMH) model [11] for Caucasians and the Japan Clinical Oncology Group (JCOG) model [14] for Asians have been the most commonly used by oncologists. The two models were developed and validated in datasets derived from clinical trials [11, 14–16]. Wang et al. [17] and Custodio et al. [18] constructed prognostic models using real-world datasets of Chinese and European patients, respectively, but only for a specific subset of gastric cancer. Custodio et al.’s [18] AGAMENON nomogram was developed in patients with Her-2 positive disease who received trastuzumab, while Wang et al.’s [17] model was for patients with good performance (PS: 0–1). Other models were criticized either for lack of validation [19–21] or because validation only involved the inner cohort [22–24]. More importantly, while applying these models in our dataset, none of them did good job in predicting survival of Chinese patients (unpublished data). Current model was derived from a dataset of non-selective populations in real clinical settings and validated in another non-selective patient cohort. Thus, our model, especially its simplified version, was expected to be more applicable and practical.

Our prognostic model included a novel set of variables that differed from the above-mentioned models. We selected laboratory parameter candidates for predictive factors in view of the following considerations: (1) convenience in detection, (2) routine clinical testing, (3) relatively steady results across complicated illnesses, and (4) patients’ cancer-related conditions. HGB and ALB levels were key parameters for nutritional status and tolerance to anticancer therapy. Low levels of HGB and ALB were associated with poor prognosis in patients with advanced gastric cancer [25, 26]. ALB has been adopted in several other prognostic models [19, 22–24]. LDH is a key enzyme in anaerobic glycolysis, which reflecting the metabolic rate of cancer to some degree. The expression of LDH was a negative prognostic indicator in gastric cancer [27, 28], but most models did not involve LDH. The CEA level is a common test in the diagnosis and monitoring of gastric cancer but is not present in existing prognostic models. Therefore, LDH and CEA levels were introduced into the current study’s analysis. More importantly, both were retained in the model.

In this study, our prognostic scoring model divided the patients into low-, intermediate- and high-risk subgroups in the development and validation cohorts. Although survival curves were separated among different subgroups, the median survival time of each risk subgroup (low-risk: 10.5 months, intermediate-risk: 5.6 months, high-risk: 2.0 months) was shorter in the validation cohort than that in the development cohort (14.4, 6.3, and 4.1 months for low-, intermediate- and high-risk subgroups, respectively). These differences were attributed to the heterogeneous baselines of the two cohorts. The patients in the validation cohort were older and had poorer performance, more G3–4 tumors, and lower median HGB concentration and ALB level. In the AGAMENON model, which was developed in Caucasian patients with advanced esophagogastric adenocarcinoma by Custodio et al. [18], the median OS for low-, intermediate -, and high-risk patients were 14, 9.4, and 5.8 months in the derivation set. These results were comparable to ours. However, in a recent Chinese cohort, advanced patients were classified by another model into low-, intermediate -, and high-risk subgroups with median OS of 19.7, 10.7, and 5.1 months, respectively [17]. While comparing the baseline characteristics of patients in the two studies, there were more patients with good performance, more patient received palliative gastrectomy and palliative chemotherapy, and less patients with poorly differentiated tumors in that cohort than those in our development cohort. All these contributed to the longer survival times.

Our nomogram showed moderate predictive capabilities. The c-indices of the nomogram were less than 0.7, partially due to the retrospective design of this study. ECOG performance status was evaluated by different clinical doctors. Images of metastasis were not independently reviewed and confirmed. The chemotherapeutic regimens were not analyzed. More important, this model did not include Lauren subtype and Her-2 status of the tumor, which were demonstrated to be linked to survival of gastric cancer patients [29, 30]. All these contributed to the moderate ability of prediction.

## Conclusion

In the current study, we developed and validated a nomogram-based prognostic scoring model, prognostic scoring model, and simplified surrogate stratified metastatic or recurrent gastric carcinoma into low-, intermediate-, and high-risk subgroups in terms of their survival. This model can be used as a tool for clinical mortality risk stratification.

## Supplementary Information

**Additional file 1. **Other clinical characteristics of gastric cancer patients in development cohort and validation cohort. This file provided the WHO histology and available information on Her-2 status of the tumor for patients in development and validation cohort.

**Additional file 2.** Frequencies of total score and simplified score for patients in development and validation cohort. Anhui Medical University (Anhui Province, China); MMH: Ma’anshan Municipal People’s Hospital (Anhui Province, China). This file provided the histograms of total score and simplified score in the two cohorts.

## Data Availability

The datasets generated during the current study are available from the corresponding author on reasonable request.

## Abbreviations

AHMU: Anhui Medical University
MMH: Ma’anshan Municipal People’s Hospital
HGB: Hemoglobin
ALB: Albumin
LDH: Lactate dehydrogenase
CEA: Carcinoembryonic antigen
HR: Hazard ratio
CI: Confidence interval
mOS: Median overall survival
PS: Performance status
ECOG: Eastern Cooperative Oncology Group
RMH: Royal Marsden Hospital
JCOG: Japan Clinical Oncology Group
