# Supplementary material for: Development and validation of a prognostic scoring model for mortality risk stratification in patients with recurrent or metastatic gastric carcinoma
Source: BMC Cancer. 2021 Dec 12;21:1326. doi: 10.1186/s12885-021-09079-7 (PMC8666033; doi:10.1186/s12885-021-09079-7)
Supplement: Supplementary file 1 — Additional file 1. Other clinical characteristics of gastric cancer patients in development cohort and validation cohort. This file provided the WHO histology and available information on Her-2 status of the tumor for patients in development and validation cohort. [file 12885_2021_9079_MOESM1_ESM.docx]

Supplementary file 1

Table. Other clinical characteristics of gastric cancer patients in development cohort and validation cohort.

| Clinical characteristics | Frequencies distribution | | | |  | Univariate Cox regression (development cohort) | | | |
| --- | --- | --- | --- | --- | --- | --- | --- | --- | --- |
|  | AHMU cohort  [*n (%)*, *n*=401] | MMH cohort  [*n (%)*, *n*=214] | *χ2* | *P* |  | *β* | *HR* | *95% CI* | *P* |
| WHO-histological subtype |  |  | 21.487 | <0.001 |  |  |  |  |  |
| Adenocarcinoma, NOS | 284 (70.8) | 159 (74.3) |  |  |  | reference | | | |
| Tubular/ Papillary adenocarcinoma | 12 (3.0) | 4 (1.9) |  |  |  | -0.752 | 0.47 | 0.22~1.00 | 0.050 |
| Mucinous adenocarcinoma | 39 (9.7) | 16 (7.5) |  |  |  | 0.420 | 1.52 | 1.07~2.15 | 0.017 |
| Signet ring cell carcinoma | 24 (6.0) | 29 (13.6) |  |  |  | -0.165 | 0.85 | 0.53~1.35 | 0.489 |
| Other type of carcinoma^a^/NOS | 42 (10.5) | 6 (2.8) |  |  |  | 0.224 | 1.25 | 0.90~1.75 | 0.190 |
| Her-2 status |  |  | 5.645 | 0.059 |  |  |  |  |  |
| Negative | 50 (12.5) | 42 (19.6) |  |  |  | reference | | | |
| Positive | 9^b^ (2.2) | 4^c^ (1.9) |  |  |  | -0.657 | 0.52 | 0.23~1.15 | 0.107 |
| Unknown | 342 (85.3) | 168 (78.5) |  |  |  | -0.151 | 0.86 | 0.63~1.18 | 0.352 |

^a^Other type of carcinoma including un-differentiated carcinoma, adenosquamous carcinoma and small cell carcinoma. ^b^5 patients received trastuzumab. ^c^1 patient received trastuzumab.

AHMU: Anhui Medical University (Anhui Province, China); MMH: Ma’anshan Municipal People’s Hospital (Anhui Province, China); HR: hazard ratio; CI: confidence interval; WHO: World Health Organization; NOS, not otherwise specified.
