# Supplementary material for: Development and validation of a prognostic scoring model for mortality risk stratification in patients with recurrent or metastatic gastric carcinoma
Source: BMC Cancer. 2021 Dec 12;21:1326. doi: 10.1186/s12885-021-09079-7 (PMC8666033; doi:10.1186/s12885-021-09079-7)
Supplement: Supplementary file 2 — Additional file 2. Frequencies of total score and simplified score for patients in development and validation cohort. Anhui Medical University (Anhui Province, China); MMH: Ma’anshan Municipal People’s Hospital (Anhui Province, China). This file provided the histograms of total score and simplified score in the two cohorts. [file 12885_2021_9079_MOESM2_ESM.pdf]

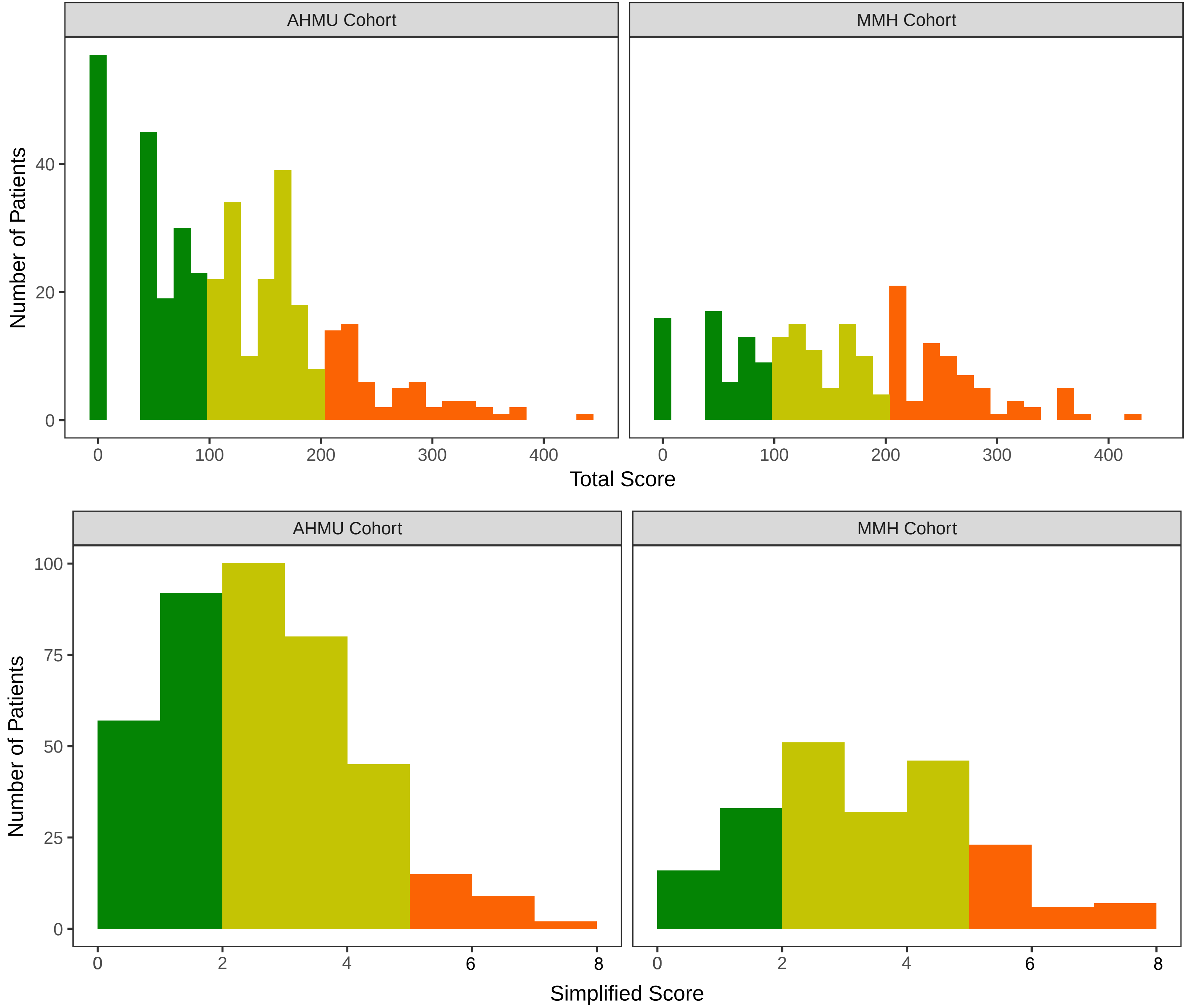

Supplementary file 2
Figure. Frequencies of total score (upper panel) and simplified score (Lower panel) for patients in development (AHMU) and validation (MMH) cohort. AHMU: Anhui Medical University (Anhui Province, China); MMH: Ma'anshan Municipal People's Hospital (Anhui Province, China).
